# A Novel Z-Ring Associated Protein ZapA-Like Protein (PA5407) From *Pseudomonas aeruginosa* Promotes FtsZ to Form Double Filaments

**DOI:** 10.3389/fmicb.2021.717013

**Published:** 2021-08-04

**Authors:** Xiaoyu Wang, Xueqin Ma, Zhe Li, Mingyue Niu, Meiting Zhai, Yaodong Chen

**Affiliations:** Key Laboratory of Resources Biology and Biotechnology in Western China, Ministry of Education, College of Life Sciences, Northwest University, Xi’an, China

**Keywords:** bacterial cell division, FtsZ, ZapA, Z-ring, FtsZ assembly, ZapA-like protein

## Abstract

Bacterial cell division is initiated by the assembly of the contraction ring (Z-ring), which consists of the self-assembled FtsZ protofilaments and dozens of other associate proteins. ZapA, a regulatory protein found in almost all bacteria, stabilizes FtsZ protofilaments to form bundles and enhances the Z-ring condensation. Here, we reported that another small protein from *Pseudomonas aeruginosa*, ZapA-Like protein (ZapAL; PA5407), is a new FtsZ associated protein. ZapAL exists in many *Pseudomonas* species and shares only 20% sequence identity to ZapA. ZapAL interacts with FtsZ and induces FtsZ to form long straight double filaments; in comparison, ZapA promotes long bundles with multiple FtsZ filaments. ZapAL has only a mild effect on GTPase activity of FtsZ, which is reduced by around 26% when 10 μM ZapAL is added in the solution. However, to study their assembly dynamics using light-scattering assay, we found that FtsZ-ZapAL double filament is stable and no depolymerization process is observed, which is different from ZapA. Further research found that ZapA and ZapL are likely to form heterodimers. The bundles formed by the mixture of FtsZ-ZapA-ZapAL will depolymerize after GTP is hydrolyzed. Consistent with ZapAL interaction with FtsZ *in vitro*, the expression of ZapAL-GFP was observed as a narrow band or spots in the middle of the cells, suggesting that it is a component of bacterial division machinery. Similar to ZapA, ZapAL is also not essential for bacterial cell division. Little changes were observed when *zapAL* gene was deleted, or overexpressed under normal conditions; however, overexpression of ZapAL caused *zapA*-deficient cells to grow approximately two times longer, showing a mild bacterial division defect. Although we still do not know the exact physiological roles of ZapAL, our results suggest that ZapAL is a novel Z-ring associate protein, which may work together with ZapA to stabilize the FtsZ protofilament and Z-ring structure.

## Introduction

Bacterial tubulin homolog FtsZ spontaneously polymerizes to dynamic protofilaments as a scaffold and combines with dozens of accessory proteins to form a highly dynamic Z-ring – a large protein complex, also known as the bacterial divisome. Z-ring is precisely and effectively activated and regulated by a variety of proteins, including scaffolders, stabilizers, regulators, and many downstream proteins and enzymes (Haeusser and Margolin, 2016; Erickson and Osawa, 2017; McQuillen and Xiao, 2020; Ur Rahman et al., 2020; Barrows and Goley, 2021). In *Escherichia coli*, FtsZ with two other proteins, FtsZ interacting protein A (ZipA) and FtsA, are the earliest proteins to assemble the proto-ring (Hale and de Boer, 1997; Pichoff and Lutkenhaus, 2002; Vicente and Rico, 2006). FtsA and ZipA are two membrane proteins that have the function of anchoring FtsZ on the inner membrane. FtsA is also a key protein for recruiting other downstream proteins. Although not essential for bacterial division, the FtsZ associated protein, ZapA, is considered as a stabilizer of the proto-ring (Huang et al., 2013). In *Bacillus subitilis*, it is reported that FtsA, SepF, ZapA, and EzrA directly interact with FtsZ and participate in the early proto-ring assembly (Gamba et al., 2009). Then, the proto-ring provides a scaffold for the binding of dozens of other associated proteins and downstream proteins.

FtsZ is the crucial protein for bacterial cell division. *In vitro*, fast-growing bacterial FtsZ usually self-assembles into mostly single, dynamic protofilaments (Chen et al., 2005; Chen and Erickson, 2005), but through lateral contact, FtsZ rearranges into bundles or sheets in the solution of calcium, high concentration magnesium, or in some crowding environments (Yu and Margolin, 1997; Mukherjee and Lutkenhaus, 1999; Chen and Erickson, 2009). Some slow-growing bacterial and chloroplast FtsZ could assemble into bundles, and circles directly (White et al., 2000; Chen et al., 2007, 2017b; Wang et al., 2019; Porter et al., 2021). FtsZ filaments are also regulated by some associated proteins. Positive regulators, so-called stabilizers, could enhance FtsZ polymerization and form bundles by increasing the lateral interaction of the FtsZ protofilaments (Huang et al., 2013; Barrows and Goley, 2021). Recent studies using super-resolution fluorescence microscopy revealed that FtsZ filaments, as well as Z-ring *in vivo*, are very dynamic, traveling with a treadmilling pattern, selectively adding FtsZ subunits to one end and releasing from another end (Bisson-Filho et al., 2017; Yang et al., 2017; Ramirez-Diaz et al., 2018; McCausland et al., 2021).

In *E. coli*, FtsZ stabilizers include ZipA and Zap family proteins (FtsZ associated protein), such as ZapA, ZapC, and ZapD (Huang et al., 2013). ZipA exists only in γ–proteobacteria. It is a transmembrane protein that can attach FtsZ filaments onto the inner membrane (Hale and de Boer, 1997). ZipA was previously considered a Z-ring stabilizer since it could promote FtsZ into bundles (Raychaudhuri, 1999; Hale et al., 2000). However, several *in vitro* studies showed that ZipA only induces FtsZ bundling when pH is below 7 (Mateos-Gil et al., 2012), and when the pH is larger than 7, ZipA has the function of enhancing and stabilizing the FtsZ curved conformation, not bundling (Chen et al., 2017a; Rahman et al., 2020). ZapA, ZapC, and ZapD are all FtsZ stabilizers, which are located at midcell and promote FtsZ to form bundles, but they do not share any primary sequence identity (Gueiros-Filho and Losick, 2002; Hale et al., 2011; Durand-Heredia et al., 2012; Huang et al., 2013). Both ZapC and ZapD mainly exist in γ–proteobacteria, and ZapA is broadly conserved in almost all bacteria. ZapA is a small cytoplasmic protein; previous results reported that ZapA dimers or tetramers cross-link adjacent FtsZ protofilaments to associate into long straight loose bundles and/or sheets *in vitro* and thus increase the stability of the Z-ring *in vivo* (Gueiros-Filho and Losick, 2002; Low et al., 2004; Mohammadi et al., 2009; Pacheco-Gomez et al., 2013; Roseboom et al., 2018; Rahman et al., 2020). Even though ZapA is functionally redundant under normal conditions, and ZapA knockout strains show minor morphological changes, previous results suggested that *zapA* deletion strain in *B. subitilis* had serious defects in bacterial division function if it is knocked out together with the *ezrA* gene, or the level of FtsZ is reduced (Gueiros-Filho and Losick, 2002). Different from FtsZ tight bundling induced by divalent cations, which significantly inhibit FtsZ GTPase activity and dynamic (Yu and Margolin, 1997; Mukherjee and Lutkenhaus, 1999; Chen and Erickson, 2009), the loose bundles or sheets promoted by ZapA has only mild reduction of FtsZ GTPase activity (Gueiros-Filho and Losick, 2002; Mohammadi et al., 2009; Rahman et al., 2020). Further studies by super-resolution fluorescence microscopy revealed that ZapA did not alter FtsZ treadmilling rates *in vivo* (Walker et al., 2020; Squyres et al., 2021) and *in vitro* (Caldas et al., 2019).

FtsZ bundling may be an important process for bacterial division. Recent research suggested that the FtsZ stabilizers increase the Z-ring condensation, beneficial for recruiting downstream proteins to the division site (Squyres et al., 2021). Several different FtsZ stabilizers were also identified in different bacterial species, including GpsB in *Staphylococcus aureus* (Eswara et al., 2018), SepH in actinobacteria (Ramos-Leon et al., 2021), WhmD in *Mycobacterium smegmatis* (Bhattacharya et al., 2017), and MsmK in *Streptococcus suis* (Tan et al., 2021).

Here, we reported a new FtsZ stabilizer: a small regulatory protein, ZapA-Like protein (ZapAL; PA5407), from *Pseudomonas aeruginosa*. ZapAL contains 96 amino acids. Although ZapAL has only about 20% sequence identity with ZapA (PA5227), it is also labeled as ZapA in some sequencing data. To solve the puzzle, we studied the properties of this protein and found that it is a novel Z-ring associated protein that can stabilize FtsZ protofilaments to form double straight filaments.

## Materials and Methods

### Bacterial Strains, Plasmids, and Growth Conditions

Bacterial strains were grown in LB or on LB agar at 37°C supplemented with the following antibiotics when necessary: 100 μg/ml Carbenicillin and 50 μg/ml Tetracycline. Liquid cultures were grown under aeration at 37°C at 250 rpm.

Plasmids and oligonucleotides used to generate or to verify strains and plasmids are listed in Tables 1 and 2, respectively.

**Table 1:** List of strains and plasmids used in this study.

Strain or plasmid	Phenotype	Source or reference
*Escherichia coli*
DH5α	*F^–^ φ80lacZ ΔM15 Δ(lacZYA-argF)U169 recA1 endA1 hsdR17(rk^–^, mk^+^)phoA supE44 thi-1 gyrA96 relA1 tonA*	Lab stock
BL21(DE3)	*F^–^ ompT gal dcm lon hsdSB*(rB- mB-) λ(DE3)	Lab stock
Plasmid
Pet15b	His6-tag expression vector, Amp^r^	Lab stock
Pet15b-*ftsz*	Pet15b containing the entire *ftsZ* gene	Chen et al., 2012
Pet15b-*zapAL*	Pet15b containing the entire *zapAL* gene	This study
Pet15b-*zapA*	Pet15b containing the entire *zapA* gene	Rahman et al., 2020
*Pseudomonas aeruginosa*
PAO1	Wild type	
Δ*zapAL*	*zapAL* knock out mutant of PAO1	This study
Δ*zapA*	*zapA* knock out mutant of PAO1	This study
Δ*zapAL*Δ*zapA*	*zapAL/zapA* knock out mutant of PAO1	This study
Plasmid
pEX18AMP	*oriT^+^sacB^+^* gene replacement vector with multiple-cloning site from pUC18, Amp^r^	Hoang et al., 1998
pME6032-*zapAL*	pME6032 containing the entire *zapAL* gene	This study
PME6032-*zapAL-gfp*	pME6032 containing the entire *zapAL* gene fused with GFP tag at C-terminal; Tc^r^	This study
pME6032-*zapA*	pME6032 containing the entire *zapA* gene	This study

**Table 2:** Primers used in this study.

Primer	Sequence (5ꞌ → 3ꞌꞌ)	Restriction site
pME-*gfp*-S	AAA GGTACC ATGAG TAAAG GAGAA GAACT	*Kpn*I
pME-*gfp*-A	AAA AGATCT TTATT TGTAT AGTTC ATCCA	*Bgl*II
pME-*zapAL-gfp*-S	AAAAGAATTCATGAGCCGCGACGGCGTCCA	*EcoR*I
pME-*zapAL-gfp*-A	AAAA GGTACC GCCGGA TGCCCC GGCGAT	*Kpn*I
pME-*zapA-gfp*-S	AAA GAATTC ATGAG CCAGT CGAAT ACCCT	*EcoR*I
pME-*zapA-gfp*-A	AAAA GGTACC GCCGGA TGCCCC GGCGAT	*Kpn*I
pME-*zapAL*-S	AAAA GGTACC ATGAG CCGCG ACGGC GTCCA	*Kpn*I
pME-*zapAL*-A	AAAA AGATCT CTAGCC GGATGC CCCGGC GAT	*Bgl*II
pEX-*zapAL*-upstream-S	ATAGGTACCAACGAACTCGTTCGGCGGCT	*Kpn*I
pEX-*zapAL*-upstream-A	GGTTCTAGATGGCTCATGTGTCCTGCTCC	*Xba*I
pEX-*zapAL*-downstream-S	AAATCTAGAGATGCCGGCGAAGCCTGACC	*Xba*I
pEX-*zapAL*-downstream-A	AGTAAGCTTTAGGGGCTGGCGGGATCCAG	*Hind*III
pEX-*zapA*-upstream-S	ATA GAATTC GTGCGCCAGATCACAAGTTA	*EcoR*I
pEX-*zapA*-upstream-A	AGG TCTAGA TGTTCTCCAGCATGATCTCG	*Xba*I
pEX-*zapA*-downstream-S	AAC TCTAGA CCTGGAAGCGCTGGTCGAGC	*Xba*I
pEX-*zapA*-downstream-A	ATA AAGCTT CTACGAGCTGGATGGCCGCC	*Hind*III

Restriction enzyme sites are underlined.

### Protein Expression and Purification

Expression vectors for FtsZ, ZapAL, and ZapA from *P. aeruginosa* were constructed in the plasmid pET15b at the NdeI/BamHI sites. After the plasmid transformed into an *E. coli* strain BL21(DE3), protein expression was induced at 16°C overnight by the addition of 0.5 mM isopropyl β-D-1-thiogalactopyranoside (IPTG). Following sonication and centrifugation, the soluble His6-tag proteins were purified by affinity chromatography on a Talon column (Clontech Lab, Inc.). Proteins were firstly washed with 0–30 mM imidazole, and then eluted with the elution buffer containing 50 mM Tris pH 7.7, 300 mM KCl, and 300 mM imidazole. After dialysis with HMK buffer (50 mM HEPES, pH 7.5, 5 mM MgAc, and 100 mM KAc), ZapAL and ZapA proteins with His6 tag were stored at −80°C.

Since FtsZ with His-tag may affect its activity, the purified His6-PaFtsZ was incubated with 2 units/ml of thrombin for 2 h at room temperature to remove the His6-tag. A further purification followed by chromatography on a source Q 10/10 column (GE healthcare) with a linear gradient of 50–500 mM KCl in 50 mM Tris, pH 7.9, 1 mM EDTA, and 10% glycerol. The purified proteins were dialyzed into HMK buffer and stored at −80°C.

### Construction of *P. aeruginosa* Deletion Mutants

A SacB-based strategy was applied to generate the *P. aeruginosa* gene knockout mutants, modified as previously described (Hoang et al., 1998; Peng et al., 2020). Briefly, upstream and downstream fragments of the genes were amplified by PCR using the corresponding primers (Table 1). Each fragment contained about 2,000 bp. After digested with appropriate restriction enzymes, both upstream and downstream fragments were ligated and inserted into pEX18Amp plasmid. To avoid errors introduced by PCR, the DNA inserts were verified by DNA sequencing. The resultant plasmids were electroporated into PAO1 and subjected to selection for carbenicillin-resistance. Colonies were then selected on *Pseudomonas* isolation agar plates with 10% sucrose, which indicates a double-crossover event and therefore gene replacement occurring. The resultant mutants were verified by PCR on the region containing the target gene.

### Kinetics Measurement

The assembly kinetics of FtsZ filaments and bundles with or without ZapA and ZapAL were measured by the light scattering method at room temperature. After FtsZ and the ZapAL or ZapA mixture is quickly mixed with GTP, the changes of the light scattering signal are tracked to obtain their assembly kinetics. Both excitation and emission were set to 340 nm with a Shimadzu RF-5301 PC spectrofluorometer. Each measurement was repeated two or three times.

### Negative Stain Electron Microscopy

Negative stain electron microscopy (EM) was used to visualize FtsZ filaments, as described previously (Rahman et al., 2020). Briefly, samples were incubated with GTP to polymerize for 1–3 min at room temperature. Then, 10 μl samples were added to a carbon-coated copper grid. After standing for about 5 s, the solution was quickly absorbed with filter papers. Grids were immediately stained with 3–5 drops of 2% uranyl acetate, and the excess droplets were dried with filter papers. Images were obtained on a Philips EM420 equipped with a CCD camera.

### Sedimentation Assay

To determine the composition of the FtsZ-ZapAL polymers, a sedimentation assay was applied, modified as previously described (Huang et al., 2018). The mixture of 15 μM FtsZ and 15 or 30 μM ZapAL were incubated with 1 mM GTP or without GTP at room temperature for 10 min and centrifuged at 80,000 rpm for 30 min at 25°C in a Beckman TLA100 rotor. The supernatant was carefully removed, and the pellet was resuspended in the same volume of buffer. The protein in the pellet and supernatant was assayed by SDS-PAGE. The ratio of supernatant and pellet was analyzed using ImageJ software (Schneider et al., 2012), and the protein concentrations of FtsZ or ZapAL in the pellet were calculated from the percentage of total protein concentration. The measurement was repeated three times.

### GTPase Activity Measurement

GTPase activity was determined by a continuous assay coupled with a GTP regeneration system, as described previously (Rahman et al., 2020). The solution included 1 mM Phosphoenolpyruvic acid monopotassium, 0.9 mM NADH, 10 units/ml pyruvate kinase and lactate dehydrogenase (Sigma-Aldrich), and 0.5 mM GTP, in HMK buffer (50 mM HEPES, pH 7.5, 5 mM MgAc, and 100 mM KAc), and a 3 mm path cuvette was used for measurement. In this assay, when a molecule of GTP is hydrolyzed to GDP, a molecule of NADH in the solution will be consumed in the subsequent reaction, and at the same time, GDP is regenerated to GTP. The GTP concentration in the solution remains constant, and it can avoid the effect of GDP accumulation on the GTPase activity in the solution. The GTP hydrolysis rate is equal to the NADH consumption rate, which is measured at room temperature using the extinction coefficient 0.00567 μM^−1^ cm^−1^ at 350 nm with an L5 UV-vis spectrophotometer (INESA analytical Instrument Co.). The hydrolysis rate was plotted as a function of FtsZ concentration, and the slope of the line above the critical concentration (Cc) represents the GTPase activity per unit FtsZ concentration. Each measurement was repeated two or three times.

### Microscopy and Image Acquisition

*Pseudomonas aeruginosa* PAO1 carrying pME6032*-zapAL* and pME6032*-zapAL-gfp* were cultured in LB with 50 μg/ml Tetracycline until OD600 value reached ~0.4, and 0.5 mM IPTG was added and induced at 37°C for 1.5 h. PAO1 and the deletion mutants were cultured in LB for 4 h at 37°C until OD600 reached ~0.5.

The cells in the agarose pad were visualized with phase contrast and fluorescence microscopy using a Leica DMI3000B fluorescence microscopy. PAO1 cell lengths were measured for each mutant, and the results were reported as the mean ± SD.

## Results

### ZapAL Interacts With FtsZ and Promotes FtsZ to Form Double Straight Protofilaments

**Figure 1:**
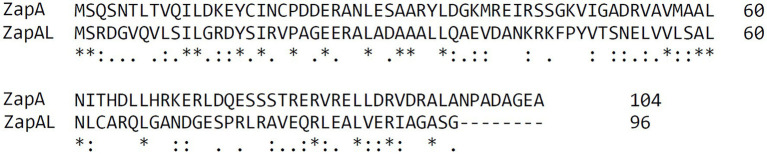
Sequence comparison of ZapA and ZapA-Like protein (ZapAL) from *P. aeruginosa* using Clustal Omega. These two proteins share only 20% sequence identity and around 40% similarity.

ZapA is a broadly conserved Z-ring associate protein in most bacteria (Gueiros-Filho and Losick, 2002). When we searched for the ZapA protein sequences in the database (Winsor et al., 2016), we found there were two small proteins from *P. aeruginosa*, PA5227 and PA5407; both were marked as ZapA in some sequencing data. We used Clustal Omega (Sievers et al., 2011) to align these two proteins and found that they share only 20% sequence identity and around 40% similarity (Figure 1). PA5227 contains 104 amino acids, which is ZapA. Another protein PA5407 contains 96 amino acids and is mainly found in *Pseudomonas* species. We name this protein ZapAL.

**Figure 2:**
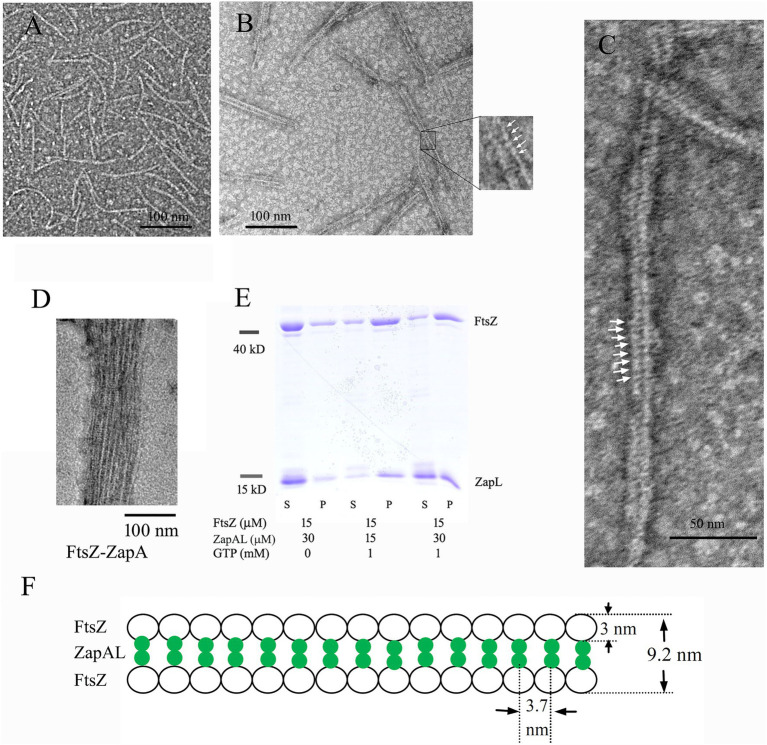
**(A–D)** show negative stain electron microscopy (EM) of 5 μM FtsZ **(A)**, 5 μM FtsZ and 10 μM ZapAL **(B,C)**, and 5 μM FtsZ and 10 μM ZapA **(D)** in the presence of 1 mM GTP. The arrows in **(B,C)** show periodic striations that could be due to ZapAL crosslinks. **(E)** shows the SDS-PAGE analysis of sedimentation in different ratios of FtsZ and ZapAL (S, supernatant; P, pellet). The concentrations of FtsZ and ZapAL in the pellet were calculated through SDS-PAGE analysis. **(F)** shows the model of FtsZ-ZapAL double filaments.

To characterize ZapAL, we first investigated whether or not ZapAL interacts with FtsZ and affects FtsZ assembly. Using negative-stain EM, we found that ZapAL induced FtsZ to form long, straight double filaments (Figure 2). FtsZ from *P. aeruginosa* alone assembles into mostly the mixture of single straight and arc-shaped filaments in the presence of GTP (Figure 2A). After ZapAL was added, FtsZ-ZapAL mostly assembles into long, double straight filaments in the presence of GTP (Figures 2B,C). It is different from ZapA which promotes FtsZ to form large bundles/sheets composed of multiple FtsZ filaments (Figure 2D, also previous publication; Rahman et al., 2020). From EM images of FtsZ-ZapAL, we could observe the striations across the FtsZ-ZapAL double filaments, indicating ZapAL cross-links (Figures 2B,C, arrows). The distance between ZapAL molecules is around 3.7 ± 0.4 nm, which suggests that ZapAL binds to each FtsZ molecule. Similar to FtsZ-ZapA, the FtsZ-ZapAL double filaments are also loose structures. The average distance from the outer edge of one FtsZ protofilament to another is about 9.2 ± 1.1 nm. Considering the thickness of FtsZ filaments in our measurement is around 3 nm, the interval distance between filaments is approximately 3.2 nm.

We next used sedimentation and SDS-PAGE to analyze the stoichiometry of the FtsZ-ZapAL copolymers (Figure 2E). Following centrifugation at 80,000 rpm, most copolymers were in pellets. Without GTP, little protein could be seen in the pellet. In the presence of GTP, FtsZ and ZapAL were pre-mixed at the ratio of 1:1 or 1:2, and the stoichiometry of the copolymer in the pellet was estimated by SDS-PAGE analysis from the percentage of total protein amounts. In the mixture of 15 μM FtsZ and 15 μM ZapAL, FtsZ concentration is around 11.5 ± 1.2 μM, and ZapAL is around 10.7 ± 1.8 μM in the pellet. In the mixture of 15 μM FtsZ and 30 μM ZapAL, FtsZ concentration is around 10.4 ± 1.5 μM, and ZapAL is around 12.6 ± 1.0 μM in the pellet. The molar ratio of FtsZ and ZapAL in the polymer is almost 1:1. We proposed a model to show the FtsZ-ZapAL double filaments (Figure 2F). Acting as a bridge, ZapAL connects two FtsZ protofilaments to form long straight double filaments.

### Characterization of the Assembly of FtsZ-ZapAL Filaments

**Figure 3:**
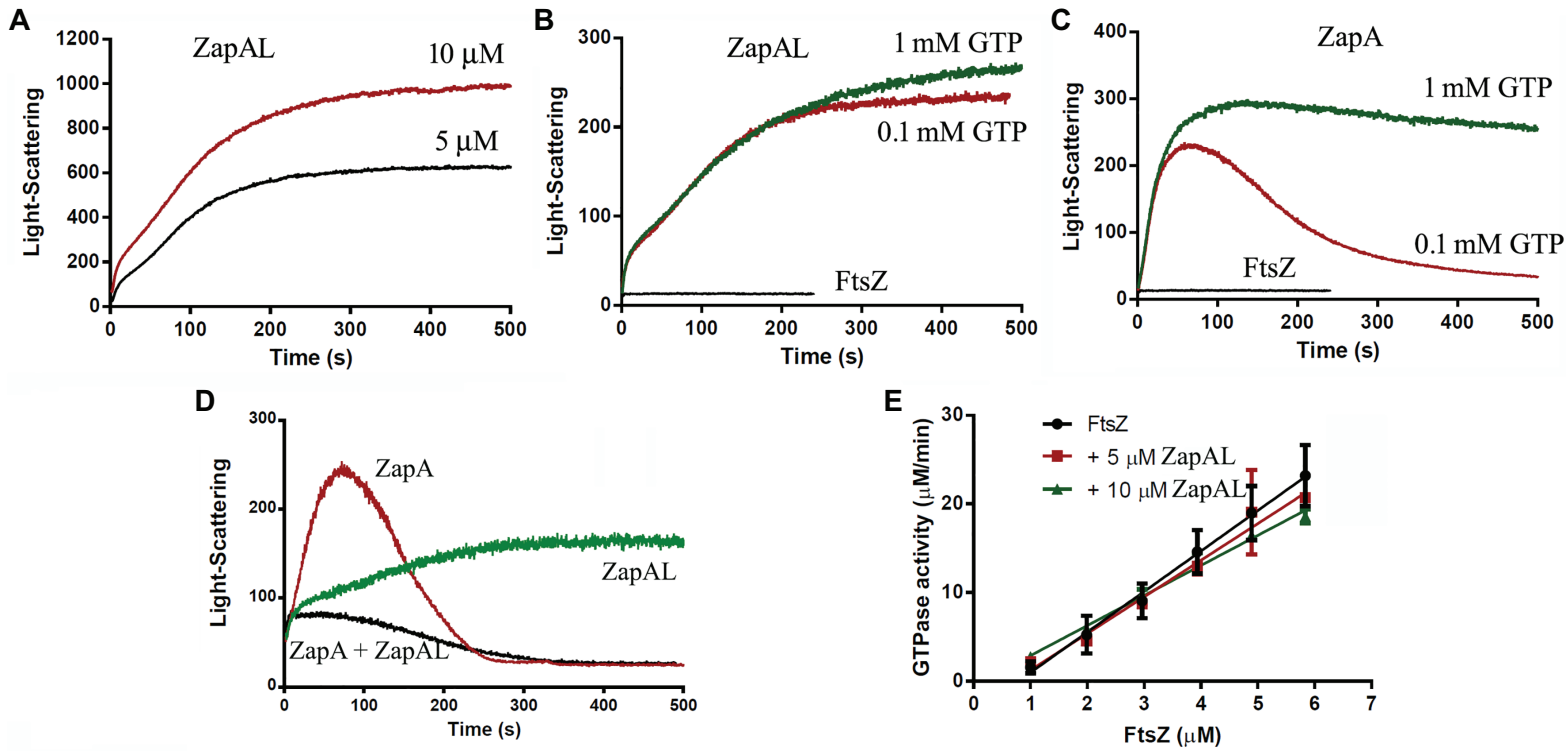
Kinetics of FtsZ assembly in the presence of or in the absence of ZapAL or ZapA followed by light scattering. **(A)** shows the bundles formation of 5 μM FtsZ-ZapAL and 10 μM FtsZ-ZapAL in HMK buffer measured by light scattering assay. **(B,C)** show the comparison of the assembly kinetics of 10 μM FtsZ-ZapAL **(B)** or 10 μM FtsZ-ZapA **(C)** in the presence of 1 mM and 0.1 mM GTP. **(D)** shows the comparison of the assembly kinetics of 10 μM FtsZ with 10 μM ZapAL, 10 μM ZapA, and the mixture of 5 μM ZapAL and 5 μM ZapA in the presence of 0.1 mM GTP. **(E)** shows the comparison of the GTPase activity of FtsZ, FtsZ with 5 μM ZapAL and FtsZ with 10 μM ZapAL at room temperature. Error bars show the SD of three replicates.

Since FtsZ-ZapAL assembles into large, straight double filaments, a light-scattering assay will be a useful tool to measure its assembly dynamics. In our experiments, we used the light-scattering assay to study how ZapAL affects the FtsZ assembly kinetics. Figure 3 shows the comparison of FtsZ assembly kinetics with ZapAL and ZapA. FtsZ alone assembles into single filaments and its light-scattering signal is very weak. ZapAL induced FtsZ to form long double filaments, corresponding to a strong light-scattering signal. Figure 3A shows the assembly of a 1:1 mixture of 5 and 10 μM FtsZ-ZapAL in the presence of 1 mM GTP. After a short fast rising, the light-scattering signal increased slowly and reached a plateau at around 300 s. However, the light-scattering signal of FtsZ-ZapAL was completely stable, and no decrease was observed in our measurements, up to 1,200 s. We repeated the measurement of 10 μM FtsZ-ZapAL with 1 mM GTP and 0.1 mM GTP, and almost similar results were obtained (Figure 3B). It is different from the assembly of FtsZ-ZapA (Figure 3C). The assembly of FtsZ-ZapA is faster; it took around 100 s to reach the plateau. Comparing the polymerization kinetics of 10 μM FtsZ-ZapA at different concentrations of GTP, we observed that the light scattering signal with 0.1 mM GTP began to decrease rapidly at about 100 s, and then decreased to near the baseline at about 250 s (Figure 3C). This shows that when GTP is used up, FtsZ-ZapA will completely depolymerize, consistent with the decrease of light scattering signal. However, it seems that the FtsZ-ZapAL copolymers are stable, and no depolymerization could be observed even with 0.1 mM GTP in our measurements (Figure 3B). The similarity between ZapAL and ZapA makes it possible to form heterodimers between them. The Figure 3D compares the effects of ZapA, ZapAL, and a mixture of ZapA and ZapAL in a ratio of 1:1 on the FtsZ polymerization kinetics in the presence of 0.1 mM GTP. We found that the light scattering intensity of the mixture of FtsZ and ZapAL-ZapA was weaker and faster, reaching the maximum in less than 20 s. Then, it began to decline, showing that it would depolymerize after GTP was hydrolyzed, similar to FtsZ-ZapA. This result implies that ZapA and ZapAL are very likely to form a heterodimer and promote FtsZ to form a small bundle, which maintain the dynamic characteristics of the FtsZ filaments.

To obtain more information, we next investigated how ZapAL affects the GTPase activity of FtsZ. Figure 3E shows the GTPase activity of FtsZ at different concentrations with or without ZapAL at room temperature. The GTPase activity of FtsZ is around 4.57 ± 0.34 GTP/FtsZ/min, and it drops to 4.14 ± 0.33 GTP/FtsZ/min with 5 μM ZapAL and to 3.39 ± 0.12 GTP/FtsZ/min with 10 μM ZapAL, only a 26% reduction. We concluded that ZapAL has a mild effect on the GTPase activity of FtsZ, similar to ZapA. If there is only 0.1 mM GTP in the solution, 10 μM FtsZ-ZapAL mixtures will hydrolyze all GTP in about 3 min; however, no depolymerization of FtsZ-ZapAL copolymers was observed in our measurements of light-scattering signals (Figure 3B), meanwhile, the filaments formed by FtsZ-ZapA and FtsZ-ZapA-ZapAL can be depolymerized after GTP is hydrolyzed.

### ZapAL Is Located in the Middle of the Cell, but It Is Not Essential for Cell Division

**Figure 4:**
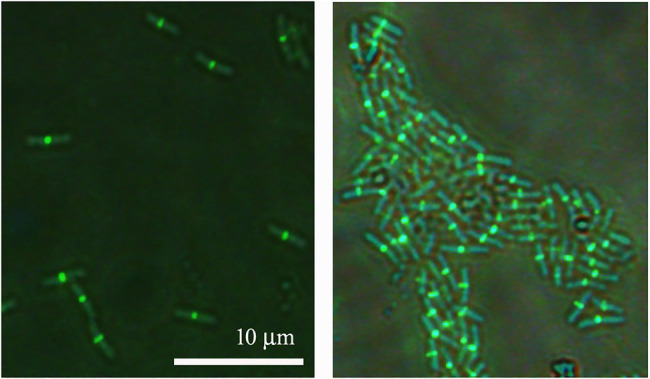
Subcellular localization of ZapAL-GFP. Fluorescence images of PAO1 cells expressing *pME6032-zapAL-gfp* are examined after 1.5 h at 37°C with 0.5 mM isopropyl β-D-1-thiogalactopyranoside (IPTG). They are two representative images from different areas of same batch of samples.

Since ZapAL interacts with FtsZ *in vitro*, we wanted to know its localization and its possible physiological function *in vivo*. Fused with GFP at C-terminus, we expressed ZapAL-GFP in wildtype PAO1 strain. Fluorescence microscopy of a GFP-ZapAL producing strain revealed narrow bands or spots in the middle of cells (Figure 4), a localization pattern characteristic of division proteins, similar to ZapA. It suggests that ZapAL is also one of the components of the division machinery.

**Figure 5:**
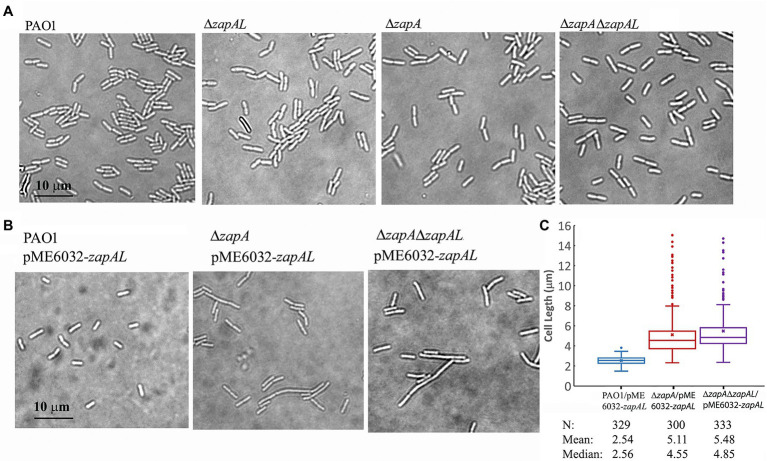
Morphology of strains of *zapAL* deletion or overexpression. **(A)** Representative images of strains of wildtype (PAO1), Δ*zapAL*, Δ*zapA*, and Δ*zapA*Δ*zapAL* show little change of cell morphology, indicating that both *zapAL* and *zapA* are not essential genes. **(B)** Overexpression of ZapAL has little effect on the morphology of wildtype PAO1, while it causes slight cell elongation in the strains of Δ*zapA* and Δ*zapA*Δ*zapAL*. **(C)** Box plot illustrating the comparison of bacterial lengths when ZapAL is overexpressed in the strains of PAO1, Δ*zapA* and Δ*zapAL*Δ*zapA*. The boxes in the box plot show the median and the first and third quartiles. The length of the upper whisker is the largest value that is no greater than the third quartile plus 1.5 times the interquartile range. The number of bacterial cells, mean and median values of bacterial length (μm) are presented at the bottom of the graph. The average length of *zapA*-deleted cells is approximately twice that of wildtype cells after overexpression of ZapAL and part of them exceed 10 μm.

To investigate the physiological function of ZapAL, we knocked out the *ZapAL* gene from *P. aeruginosa* PAO1 strain. However, we found that knockout *ZapAL* exhibited no recognizable defects in bacterial cell division and growth. Similar results were obtained after we knocked out the *zapA* gene. Since both protein ZapAL and ZapA show a function to stabilize FtsZ protofilaments, we knocked out both of the *zapA* and *ZapAL* genes, and there were still no obvious effects on bacterial morphology (Figure 5A). These results suggest that both ZapAL and ZapA are not essential for cell division under normal laboratory conditions.

In an attempt to uncover a role for ZapAL in bacterial division and growth, we also investigated the effects of the overexpressed ZapAL protein on bacterial cell division. Induced by 0.5 mM IPTG, the ZapAL protein was expressed in *P. aeruginosa* PAO1 containing *pME6032-zapAL* plasmid. However, few changes were observed when ZapAL was overexpressed (Figure 5B). But when ZapAL was overexpressed in the *zapA* knockout strain, we observed an obvious increase in bacterial length (Figure 5B). After overexpression of ZapAL, the bacterial length is around 2.5 ± 0.4 μm (*n* = 329) in wildtype PAO1 strain, 5.1 ± 2.2 μm (*n* = 300) in *zapA* deletion strain, and 5.5 ± 2.1 μm (*n* = 333) in both *zapA* and *zapAL* deletion strain (Figure 5C). It suggests that overexpression of ZapAL would mildly impair bacterial cell division without ZapA. Meanwhile, overexpression of ZapA in PAO1 strain and *zapAL* deletion has no obvious effect on bacterial division and growth under normal conditions (data not shown).

## Discussion

In this study, we reported a novel FtsZ/Z-ring associated protein ZapAL (PA5407) from *P. aeruginosa*. ZapAL is a small ZapA-like protein that contains 96 amino acids and shares around 20% identity (~ 40% similarity) with ZapA (PA5207, 104 amino acids). Similar to ZapA, ZapAL cross-links adjacent FtsZ protofilament subunits and stabilizes FtsZ polymers. However, unlike ZapA, which promotes FtsZ to form loose bundles or sheets of multiple straight filaments, ZapAL induces FtsZ to form loose, double straight filaments. *In vivo*, ZapAL-GFP is located at midcell, and it suggests that ZapAL is a Z-ring associated protein to stabilize the Z-ring. Our research revealed that ZapAL has only a mild effect on the GTPase activity of FtsZ. Approximately, 75% of GTPase activity of FtsZ can still be maintained after adding 10 μM ZapAL in solution. However, from our light-scattering experiments, we found that the FtsZ-ZapAL double filament is stable. Although GTP is required for their polymerization, the double filaments do not depolymerize after GTP is used up. Furthermore, ZapAL and ZapA are likely to form heterodimers. The mixture of ZapAL and ZapA can promote FtsZ to form a smaller bundle, which corresponds to their weaker light scattering signal. Meanwhile, like ZapA, ZapA-ZapAL heterodimer can also maintain the dynamic characteristics of FtsZ protofilaments, and the bundles they form can be depolymerized after GTP hydrolysis. This implies that ZapAL and ZapA together play a physiological function of stabilizing the Z-ring *in vivo*.

From our studies on the biochemical properties of ZapAL, an interesting finding is a contradiction between its mild reduction in FtsZ GTPase activity and the formation of the stable FtsZ-ZapAL double filaments. This contradiction was also found in previous studies of ZapA, and we suggested that it is due to the loose bundles or sheets formed by FtsZ-ZapA. Bundle formation usually reduces their dynamics, as well as their GTPase activity. However, the assembly of FtsZ-ZapA bundles only mildly reduced the GTP hydrolysis activity of FtsZ by less than 20%, suggesting that this loose bundle has a slight effect on the FtsZ GTPase activity, although it still affects its subunits exchange rate. FtsZ-ZapA bundle is dynamic and depolymerizes after GTP is hydrolyzed. The subunits exchange rate of the FtsZ-ZapA bundles was about four times slower than that of FtsZ protofilaments (Rahman et al., 2020). It is different from the tight bundles caused by divalent ions, which will greatly reduce the activity of FtsZ GTPase and slow down their dynamics (Yu and Margolin, 1997; Mukherjee and Lutkenhaus, 1999; Chen and Erickson, 2009). Recent research discovered that ZapA does not affect the FtsZ treadmilling rate *in vivo* and *in vitro* and suggested the dynamics of FtsZ polymers *in vivo* may be intrinsic to the polymer itself (Caldas et al., 2019; Walker et al., 2020; Squyres et al., 2021). The double filament formed by FtsZ-ZapAL also has a loose structure; the gap between two FtsZ filaments is about 3 nm. Similar to ZapA, ZapAL has only a mild reduction in the GTP hydrolysis activity of FtsZ, by about 26%. However, the FtsZ-ZapAL double filament is stable, and no depolymerization process was observed after GTP hydrolysis in our light-scattering experiments, implying that the GTP/GDP exchanges should occur within FtsZ filaments, and no depolymerization of the FtsZ filaments is required. Bridging by FtsZ associated proteins, adjacent FtsZ filaments form straight loose bundles with large gaps, which may be sufficient to allow the efficient exchange of nucleotides between the FtsZ filaments.

*Pseudomonas aeruginosa* contains two similar proteins, ZapA and ZapAL, as FtsZ stabilizers to enhance its lateral contact, suggesting that the FtsZ stabilizers *in vivo* may be important for bacterial cell division. In cells, there may be multiple FtsZ bundling proteins with overlapping functions, so knocking out a single stabilizer might lack an obvious effect on cell division under normal conditions. In *B. subtilis*, previous studies showed that FtsZ binding proteins contain ZapA, SepF, and EzrA, and knockout of *zapA* or *sepF* gene alone did not alter cell morphology, but when *ezrA* is knocked out together, the cell displays severe division impairment (Gueiros-Filho and Losick, 2002). Squyres et al. (2021) suggested that FtsZ binding proteins bundle FtsZ filaments into a condensed Z-ring, which is important to recruit downstream proteins, including cell wall synthesis enzymes to the division site. Without FtsZ-binding proteins, ZapA and EzrA, Z-ring condensation disappears. Furthermore, FtsZ suppressor mutant K86E that enhances its lateral interactions partially restores Z-ring condensation (Squyres et al., 2021). A variety of specific FtsZ bundling proteins were recently reported in different bacterial species (Bhattacharya et al., 2017; Eswara et al., 2018; Ramos-Leon et al., 2021; Tan et al., 2021), these also indicate the importance of FtsZ stabilizers for bacterial division.

Different from ZapA, our studies found that FtsZ-ZapAL double straight filaments are stable, so we initially hypothesized that ZapAL might inhibit the dynamics of FtsZ filaments and Z-ring. But, when we overexpressed ZapAL *in vivo*, we did not observe any inhibition of bacterial division under normal conditions. Interestingly, when we overexpressed ZapAL in the *zapA* deficient strains *in vivo*, we observed a mild inhibition, and the size of bacteria was twice as long as normal bacteria. It shows that ZapA may be able to competitively regulate the effects of ZapAL. This also suggests that ZapAL and ZapA may play a physiological function together, or/and ZapAL regulates the function of ZapA to maintain the stability and dynamics of the Z-ring. Further research is needed to solve this question.

## Data Availability Statement

The original contributions presented in the study are included in the article/supplementary material, further inquiries can be directed to the corresponding author.

## Author Contributions

YC conceived and designed the experiments. XW, XM, ZL, and YC did most of the experiments. MN and MZ contributed to experimental work and interpretation. YC wrote the manuscript with contributions from all authors. All authors contributed to the article and approved the submitted version.

## Conflict of Interest

The authors declare that the research was conducted in the absence of any commercial or financial relationships that could be construed as a potential conflict of interest.

## Publisher’s Note

All claims expressed in this article are solely those of the authors and do not necessarily represent those of their affiliated organizations, or those of the publisher, the editors and the reviewers. Any product that may be evaluated in this article, or claim that may be made by its manufacturer, is not guaranteed or endorsed by the publisher.
